# ATP-dependent conformational change in ABC-ATPase RecF serves as a switch in DNA repair

**DOI:** 10.1038/s41598-018-20557-0

**Published:** 2018-02-01

**Authors:** Qun Tang, Yan-Ping Liu, Hai-Huan Shan, Li-Fei Tian, Jie-Zhong Zhang, Xiao-Xue Yan

**Affiliations:** National Laboratory of Biomacromolecules, CAS Center for Excellence in Biomacromolecules, Institute of Biophysics, Chinese Academy of Sciences, Beijing, 100101 China

## Abstract

RecF is a principal member of the RecF pathway. It interacts with RecO and RecR to initiate homologous recombination by loading RecA recombinases on single-stranded DNA and displacing single-stranded DNA-binding proteins. As an ATP-binding cassette ATPase, RecF exhibits ATP-dependent dimerization and structural homology with Rad50 and SMC proteins. However, the mechanism and action pattern of RecF ATP-dependent dimerization remains unclear. Here, We determined three crystal structures of TTERecF, TTERecF-ATP and TTERecF-ATPɤS from *Thermoanaerobacter tengcongensis* that reveal a novel ATP-driven RecF dimerization. RecF contains a positively charged tunnel on its dimer interface that is essential to ATP binding. Our structural and biochemical data indicate that the Walker A motif serves as a switch and plays a key role in ATP binding and RecF dimerization. Furthermore, Biolayer interferometry assay results showed that the TTERecF interacted with ATP and formed a dimer, displaying a higher affinity for DNA than that of the TTERecF monomer. Overall, our results provide a solid structural basis for understanding the process of RecF binding with ATP and the functional mechanism of ATP-dependent RecF dimerization.

## Introduction

The ATP-binding cassette (ABC)-ATPase widely exists in all kinds of organisms. ABC-ATPase domains are found not only in ABC transporters but also in some DNA repair proteins, such as Rad50, SMC protein, MutS and UvrA^1–4^. RecF contains the conserved Walker A motif, Walker B motif and signature motif of ABC-ATPase that exhibits ATP-dependent dimerization; additionally, the signature motif residues interact with the ATP bound to the opposite molecule^5,6^.

RecF is a multifunctional protein involved in recombinant DNA repair, homologous genetic recombination, and DNA replication^7–9^. Similar to RecR and RecO, RecF is a recombination mediator protein (RMP) in the RecFOR pathway for ssDNA gap repair^10,11^. RMPs stimulate ssDNA hand-off from SSBs to RecA-like recombinases, and activate DNA repair at the damage site^12,13^. In the RecFOR pathway, RecR binds to RecO; the RecOR complex displays a high affinity for ssDNA and RecO interacts with SSBs^12,14–17^. In presence of ATP and DNA, RecF can interact with RecR in the cases of *E*. *coli* and *D*. *radiodurans*^6,18–20^; the RecOR complex is implicated in the recognition of dsDNA-ssDNA junctions, when associated with RecF^21–23^. Then, the complex facilitates RecA filament formation on SSB-coated ssDNA^24–26^.

Recombinases and RMPs are evolutionarily conserved, such as RecA, RecF and RecO in prokaryotic cells, UvsX and UvsY in Phage and Rad51 and Rad52 in eukaryotic cells^27^. The RecF structure is highly homologous to the head domain of Rad50, including α-helices from which the long coiled-coil domain of Rad50 originates. This observation implies a conserved mechanism of DNA binding and recognition of the boundaries of dsDNA regions by both proteins^1,5^.

ATP-dependent dimer assembly is essential for ABC-ATPase function, and in ABC-ATP transporters; ATP controls the engagement/disengagement of the two ABC-ATPase domains to drive the transport process^28^. Walker A motif, Walker B motif and the signature motif are conserved as ATP binding motifs belonging to the ABC transporters and ABC family of DNA-repair enzymes^29^. The Walker A motif binds the α- and β-phosphate of ATP, the Walker B motif provides the catalytic glutamate, and the signature motif pins and orients ATP during hydrolysis^30^. In the structures of Rad50, the serine residue in the signature motif is responsible for protein dimerization upon ATP binding with the Walker motifs^1,6^, Moreover, the structure of the SMC ATPase, shows that the signature motif interacts with ATP and is involved in ATP hydrolysis^2^. The biochemical study of RecF from *E*. *coli* and *D*. *radiodurans* (ECRecF and DRRecF) demonstrate that the Walker A motif binds with ATP and the signature motif mediates ATP-dependent dimerization^5,6^. At present, only the crystal structure of the DRRecF monomer has been solved^5^, and the assembly pattern of the RecF binding ATP is poorly understood. This concern is a key problem in the study of the RecFOR pathway.

To understand how the ATP-dependent RecF dimerization contributes to RecF functions, we determined a crystal structure of TTERecF-ATP at 3.0 Å resolution and characterized a series of ATP-binding sites using biochemical assays. Furthermore, we have characterised DNA-protein interaction regulated by the RecF ATP-dependent dimerization. Our study provides the molecular mechanisms of RecF ATP-driven dimerization and novel insights into RecF function.

## Results

### Crystal structure of TTERecF

To gain insight into the mechanism of ABC-ATPase RecF, we solved the crystal structures of TTERecF, TTERecF-ATP, and TTERecF-ATPɤS (Table 1). The results of size-exclusion chromatography demonstrated that in solution, TTERecF (42 KDa) forms a monomer, whereas TTERecF-ATP (84 KDa) and TTERecF-ATPɤS (84 KDa) form a dimer (Supplementary Fig. 1). TTERecF, TTERecF-ATP, and TTERecF-ATPɤS display different structural conformations.

**Table 1:** Data collection and refinement statistics.

	RecF-free	RecF-ATP	RecF-ATPɤS
**Data collection**			
Space group	C2221	P212121	C121
**Cell dimensions**			
*a*, *b*, *c* (Å)	49.8, 95.8, 167.5	108.1, 138.8, 179.6	166.7, 48.1, 116.9
α,β,γ (°)	90, 90, 90	90, 90, 90	90, 100, 90
Resolution (Å)	20–2.2 (2.24–2.20)	20–3.0 (3.05–3.00)	20–2.1 (2.14–2.10)
*R*_sym_ or *R*_merge_	15.2 (61.0)	13.0(80.3)	6.1(52.5)
*I*/ σ*I*	60.7 (12.4)	28.2(2.6)	31.2(3.0)
Completeness (%)	99.9 (100)	99.9(99.9)	99.6(99.1)
Redundancy	14.5(14.8)	8.8(8.5)	7.6(7.6)
**Refinement**			
Resolution (Å)	20–2.2	20–3.0	20–2.1
No. reflections	20,780	54,453	46688
*R*_work_/*R*_free_ (%)	17.5/22.3	19.5/25.4	17.6/21.9
**No. atoms**			
Protein	3001	11586	6082
ATP/ATP ɤS		124	31
Water	207	213	381
**Average** ***B*** **-factors**			
Protein	34.7	86.0	51.8
ATP/ATPɤS		73.1	63.4
Water	38.5	79.1	50.4
**R.m.s. deviations**			
Bond lengths (Å)	0.010	0.010	0.010
Bond angles (°)	1.26	1.44	1.27

*Number of xtals for each structure should be noted in footnote. *Values in parentheses are for highest-resolution shell.

**Figure 1:**
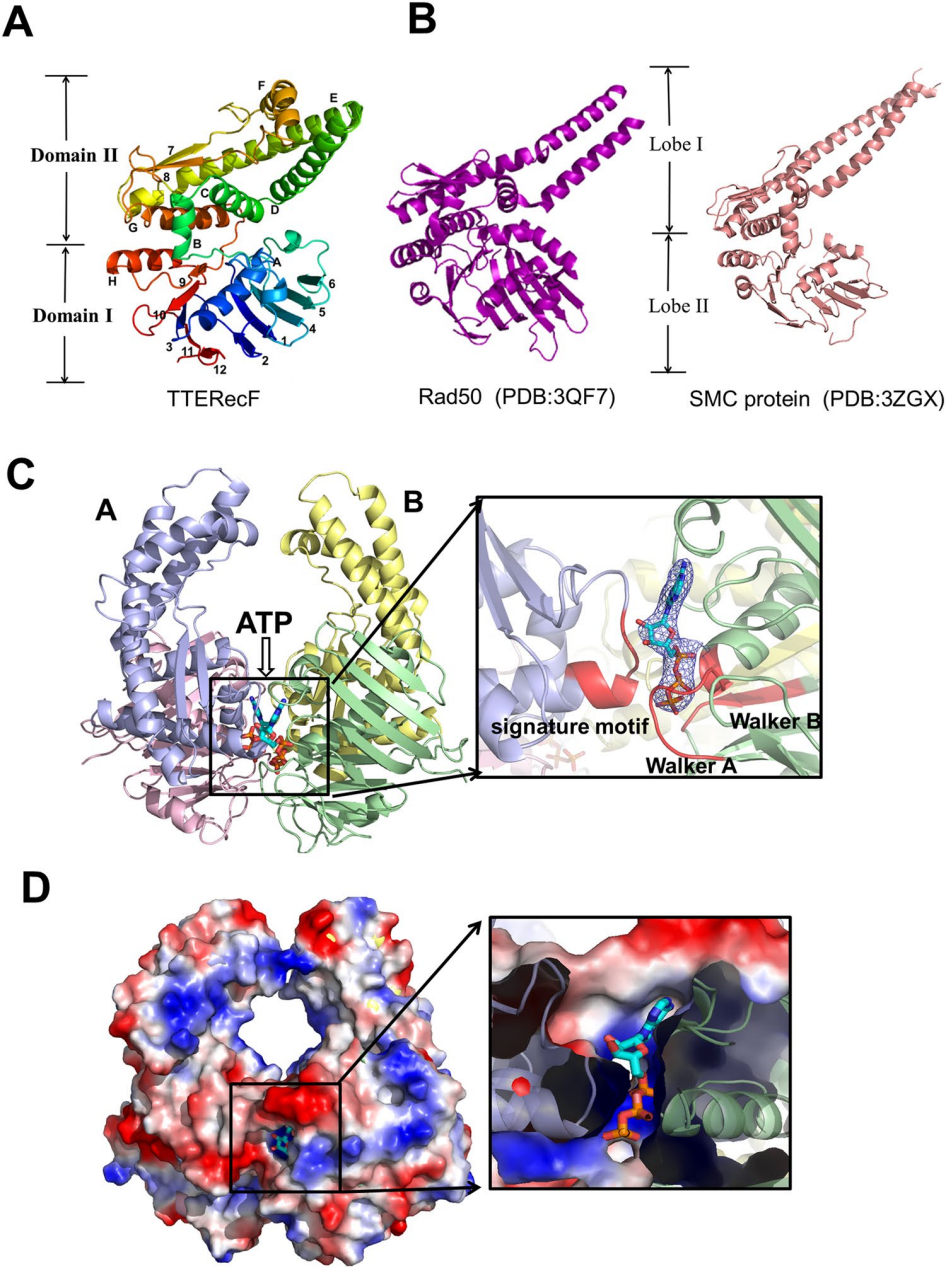
Crystal structures of TTERecF and TTERecF-ATP. (**A**) Cartoon representation of TTERecF. β-strands are numbered and α-helixes are lettered. The labels for domains I and II are indicated on the left. **(B)** Cartoon representations of the Rad50 (PDB:3QF7) and SMC protein (PDB:3ZGX) are shown in purple and salmon, respectively; the labels for lobes I and II are indicated in the middle. **(C)** The substrate ATP binds to TTERecF; TTERecF is shown as cartoon and the substrate is represented by sticks. Domains I and II are shown in pink and light blue, respectively, in the TTERecF A molecule, but green and yellow, respectively, in the TTERecF B molecule. The Walker A motif, Walker B motif, and the signature motif in the active site are shown in red. The 2*F*_*O*_- *F*_*C*_ electron density for ATP contoured at the 1.0σ level is shown as blue mesh. **(D)** Electrostatic properties of TTERecF and sliced surface view of the ATP binding tunnel. The complexes are shown as solvent-accessible surfaces coloured by electrostatic potential (red, acidic; blue, basic).

A structure-based sequence alignment between RecF orthologs reveals the conserved Walker A motif, Walker B motif and signature motif (Supplementary Fig. 2). The crystal structure of TTERecF was solved at 2.2 Å resolution through molecular replacement by using DRRecF (PDB code: 2O5V) as a model. The structure of TTERecF consisted of two domains (Fig. 1A). The ATPase domain I, which included Walker A (β3-αA) and Walker B (β9-αH), was similar to the Lobe I subdomain of Rad50 (PDB code: 3QF7) and SMC protein (PDB code: 3ZGX) head domain. Domain II contained six α-helices (αB, αC, αD, αE, αF, and αG) and two β-sheets (β7 and β8), in which the signature motif was the loop between β8 and αG. Domain II is similar to Lobe II subdomain of Rad50 and SMC protein; however, in the Rad50 and SMC proteins, helices corresponding to αD and αE extended into a long coiled-coil region, which was absent in TTERecF (Fig. 1B**)**.

### Crystal structure of TTERecF-ATP

We determined the crystal structure of the TTERecF-ATP complex at 3.0 Å through molecular replacement by using TTERecF monomer as a model. In the TTERecF-ATP structure, four RecF monomers formed two dimers in the asymmetric unit (Supplementary Fig. 3). For each stabilized dimer, ATP acted as a fastener that linked domain I of one monomer and domain II of the other monomer and was located between RecF molecules directly opposite to each other (Fig. 1C). The contact surface area between the two molecules of the dimer was 5863 Å^2^, which was 17.8% of the total dimer surface area (32919 Å^2^). The result of protein interface analysis suggested that the structures existed as stable dimers. In addition, the two long α-helices (αD and αE) of the domain II of two TTERecF formed a channel, within which numerous positive charges and hydrophobic residues were distributed (Fig. 1D). The diameter of this channel was approximately 20 Å, nearly similar to the dsDNA diameter. Thus, we speculated that DNA binds to this region, and RecF ATP-dependent dimerization is the structural foundation of DNA binding.

### Interaction between RecF and ATP

**Figure 2:**
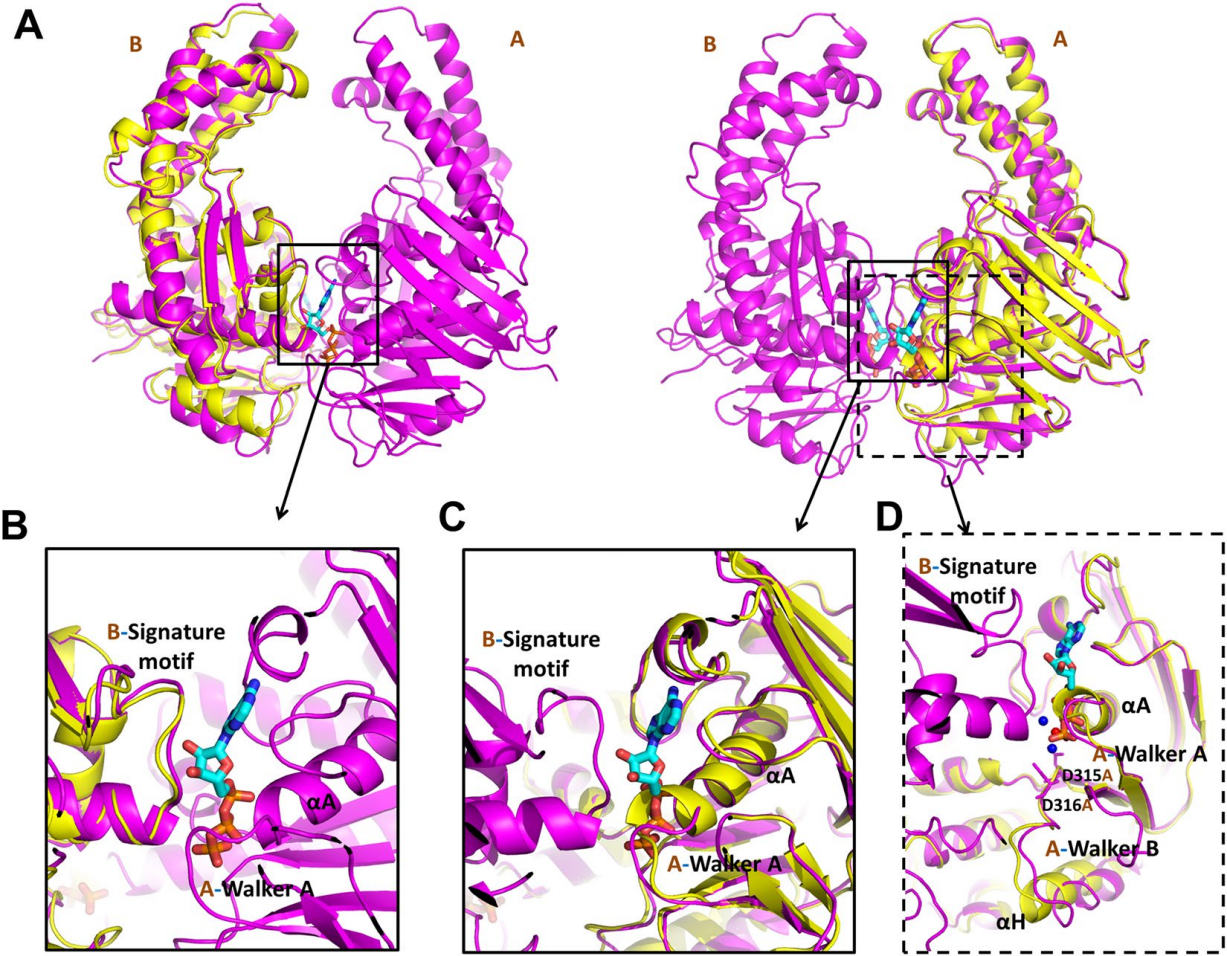
Active sites in TTERecF for binding with ATP. **(A)** Superimposed structures of TTERecF (yellow) and TTERecF-ATP complex (magenta) are shown as a cartoon model, whereas ATP is shown as sticks. RecF was respectively superimposed with the two monomers of the RecF-ATP dimer. The magnified view of the indicated regions displays the conformational change in the **(B)** signature motif, **(C)** Walker A motif, and **(D)**Walker B motif. The Mg^2+^ (red) and H_2_O molecules (blue) in the active site are indicated by circles.

Two ATP molecules were buried in the positively charged tunnel of the RecF dimer interface (Fig. 1D). ATP interacted with the Walker A motif of one TTERecF and with the conserved signature motif of the other TTERecF (Fig. 1C). Mg^2+^ in the active site binded to ATP β- and ɤ-phosphate O’s and two H_2_O molecules. One of the water molecules bonded to Asp314 and Asp315 in the Walker B motif (Fig. 2D).

**Figure 3:**
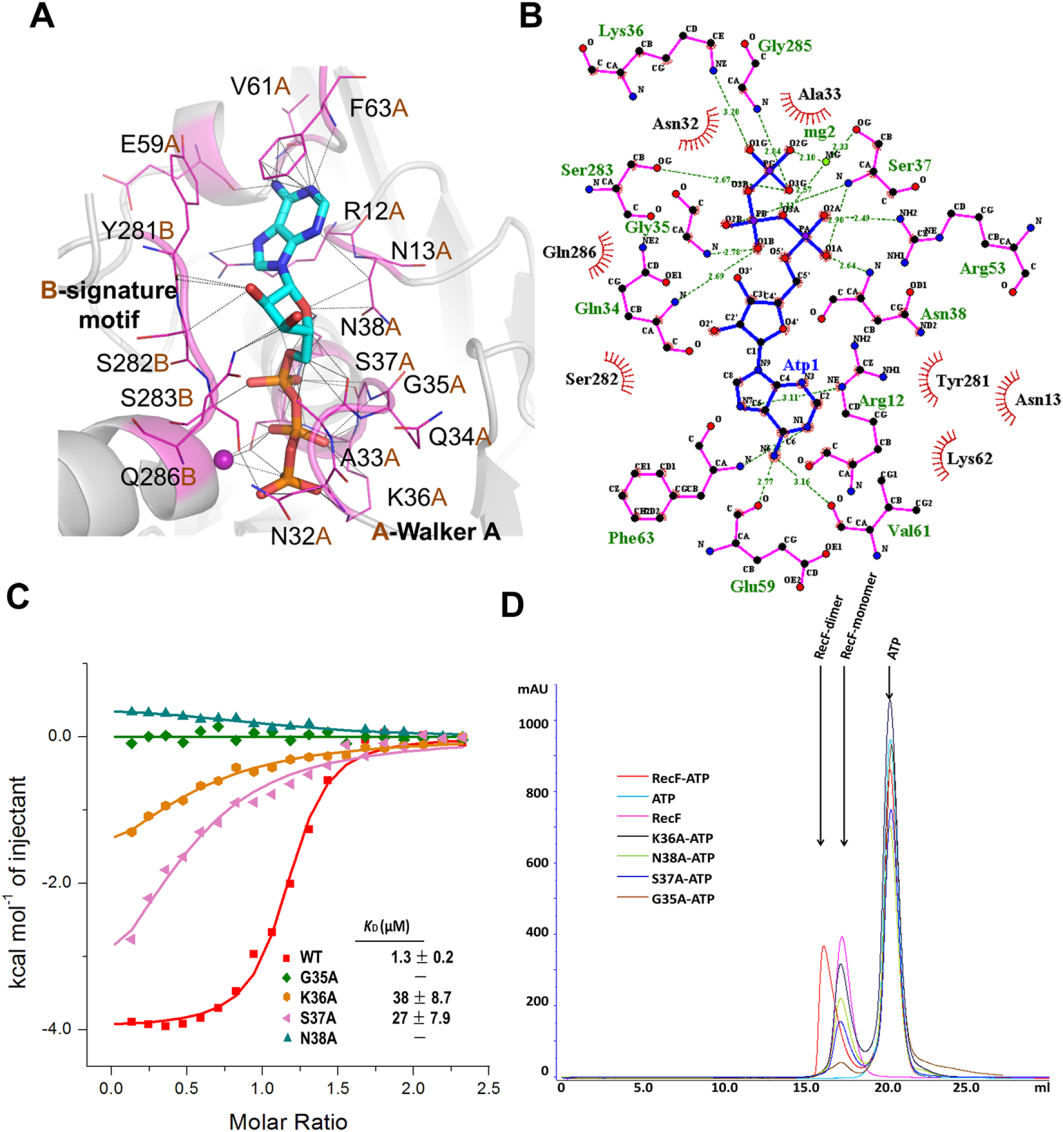
Walker A motif is essential to ATP binding and ATP-dependent dimerization. **(A)** ATP interacts with the residues in the Walker A motif and signature motif of RecF. All of the interacting residues are represented by sticks (magenta). **(B)** Extensive interactions between TTERecF and ATP. The plots were generated using LIGPLOT^33^. RecF residues and ATP are shown in pink and blue, respectively. H bonds are indicated by dashed lines (green). **(C)** ITC curves of TTERecF and the mutants of Walker A motif titrated into ATP. The ITC experiments involved 20 injections of 2 µL 1 mM ATP into 300 µL 70 µM RecF native or the mutants. **(D)** Size-exclusion chromatography analysis of TTERecF, TTERecF-ATP, ATP, G35A-ATP, K36A-ATP, S37A-ATP and N38A-ATP. Size exclusion chromatography was performed using a HiLord 16/60 Superdex 200 column (GE Health Life Sciences) at 0.5 ml/min in 50 mM Tris-HCl pH 7.0 and 300 mM NaCl. Protein elution was monitored by measuring the absorbance at 280 nm.

Comparison of the structures of the ATP-free TTERecF and one monomer of the ATP-bound TTERecF dimer showed that the two monomers were similar (Fig. 2A). The root mean square deviation (r.m.s.d) was 1.015 Å under Cα trace superimposition. Notably, two structures differed in terms of their conservative Walker A motif and Walker B motif (Fig. 2B–D). In ATP-free TTERecF, the last turn (residues 35–38) of the helix αA in the Walker A motif occupied the ATP binding site, whereas in the binding of ATP with TTERecF, the last turn was flipped by 180° relative to the ATP-free TTERecF to create a space for ATP to further interact with TTERecF (Fig. 2C). The Walker B motif residues 315 and 316 of TTERecF-ATP interacted with H_2_0, which in turn interacted with the active Mg^2+^. Moreover, the helix αH of Walker B motif moved by 4 Å relative to the ATP-free TTERecF (Fig. 2D). In addition, the conserved A loop included Phe63, which provided an aromatic side chain that was packed against the purine ring of adenine (Fig. 3A).

### Identification of RecF-ATP interaction sites

RecF contains the conserved motifs (signature motif, Walker A motif, and Walker B motif) of ABC-ATPase; the Rad50 and SMC proteins share a common mechanism and forms a functional superfamily^29^. We superimposed the ATP and the surrounding structures of the RecF and Rad50 dimers, and the Walker A motif and signature motif did not show significant change (Supplementary Fig. 4). The signature motif was essential in ATP binding, and Ser783 in the signature motif played an important role in the Rad50 dimerization^1,31^. In TTERecF, the Ser283 residue in the signature motif interacting with ATP was conserved with Ser783 of Rad50 (Fig. 3A,B). However, the conformational change in Walker A in TTERecF appeared important in ATP binding and dimerization (Fig. 2C). We used ITC to detect the dissociation constant (*K*_D_) of all mutants that interacted with ATP in the Walker A motif and signature motif. The ATP-binding ability of the conserved mutant residues S282A, S283A, and Q286A in signature motif was similar to that of the native TTERecF (Supplementary Fig. 5). The mutants G35A and N38A hardly interacted with ATP, whereas the mutants K36A and S37A displayed a considerably lower ATP binding ability than that of the native TTERecF (Fig. 3C). Size-exclusion chromatography analysis demonstrated that these four mutants cannot form a dimer in the solution (Fig. 3D). The structural and biochemical results therefore showed that Walker A motif plays an essential role in ATP binding and RecF dimerization.

### ATP-dependent RecF dimer displays high DNA affinity

**Figure 4:**
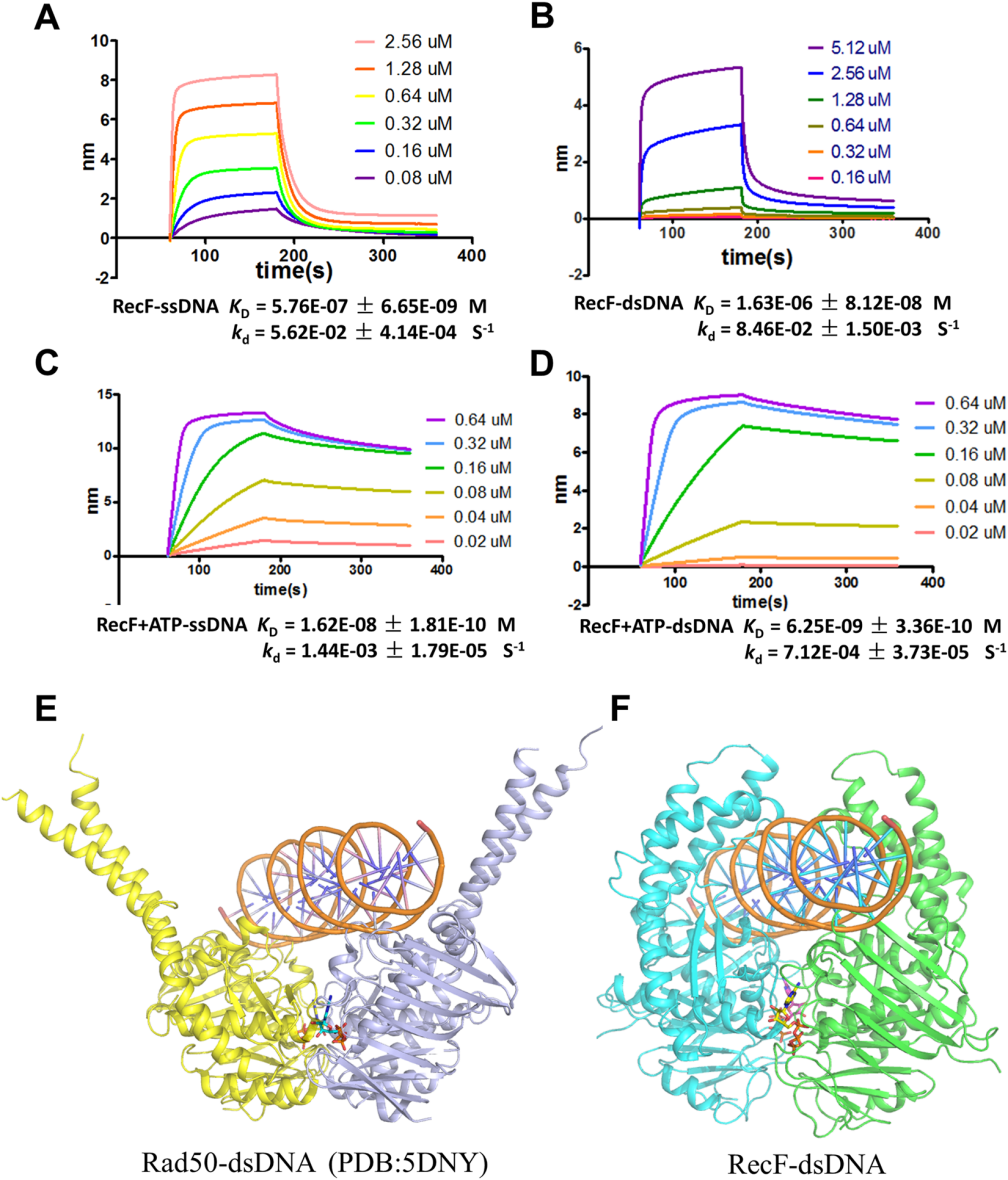
Model of RecF with dsDNA. BLI analysis of **(A)**TTERecF and **(C)**TTERecF-ATP dimer interaction with ssDNA at 25 °C. BLI analysis of **(B)** TTERecF and **(D)** TTERecF-ATP dimer interaction with dsDNA at 25 °C. Sensorgrams are shown for different concentrations of TTERecF monomer or TTERecF-ATP dimer injected over 21 mer ssDNA or 21-mer dsDNA coupled streptavidin biosensors. The apparent *K*_D_ values were calculated from the kinetic *K*_D_ (M) = *k*_d_/*k*_a_. **(E)** Cartoon representations of Rad50-dsDNA (PDB:5DNY). **(F)** Speculated structural model of RecF with dsDNA.

ATP-dependent protein dimerization is a key step in regulating the function of all ABC ATPases^30^. We used the BLI method to determine the DNA binding affinities of TTERecF. The *K*_D_ values of the TTERecF monomer interacting with the 21mer ssDNA and dsDNA were 576 nM and 1.63 µM, respectively (Fig. 4A,B), however, the TTERecF-ATP dimer binding 21mer ssDNA and dsDNA showed *K*_D_ values of 16 nM and 6.2 nM, respectively (Fig. 4C,D). The DNA-binding affinity of the TTERecF-ATP dimer became stronger than that of the TTERecF monomer. In particular, the TTERecF-ATP dimer for the dsDNA binding affinity was almost 1000 times stronger than the TTERecF monomer. Moreover, the dissociation rate (*k*_d_) also achieved a significant changes, as detected by the BLI assay. The dissociation rate of the TTERecF-ATP dimer binding with ssDNA or dsDNA (*k*_d_ = 1.44E-03 S^−1^ and 7.12E-04 S^−1^; Fig. 4,D) was lower than the TTERecF monomer binding with ssDNA or dsDNA (*k*_d_ = 5.62E-02 S^−1^ and 8.46E-02 S^−1^; Fig. 4A,B). This result illustrated that the TTERecF-ATP dimer can highly and stably bind to DNA. On the basis of the structure of the Rad50-dsDNA complex (Fig. 4E), we proposed a model of RecF interaction with dsDNA. Thus the dsDNA was precisely located in the channel of RecF dimer (Fig. 4F; Supplementary Fig. 7).

### Crystal structure of TTERecF-ATPɤS

**Figure 5:**
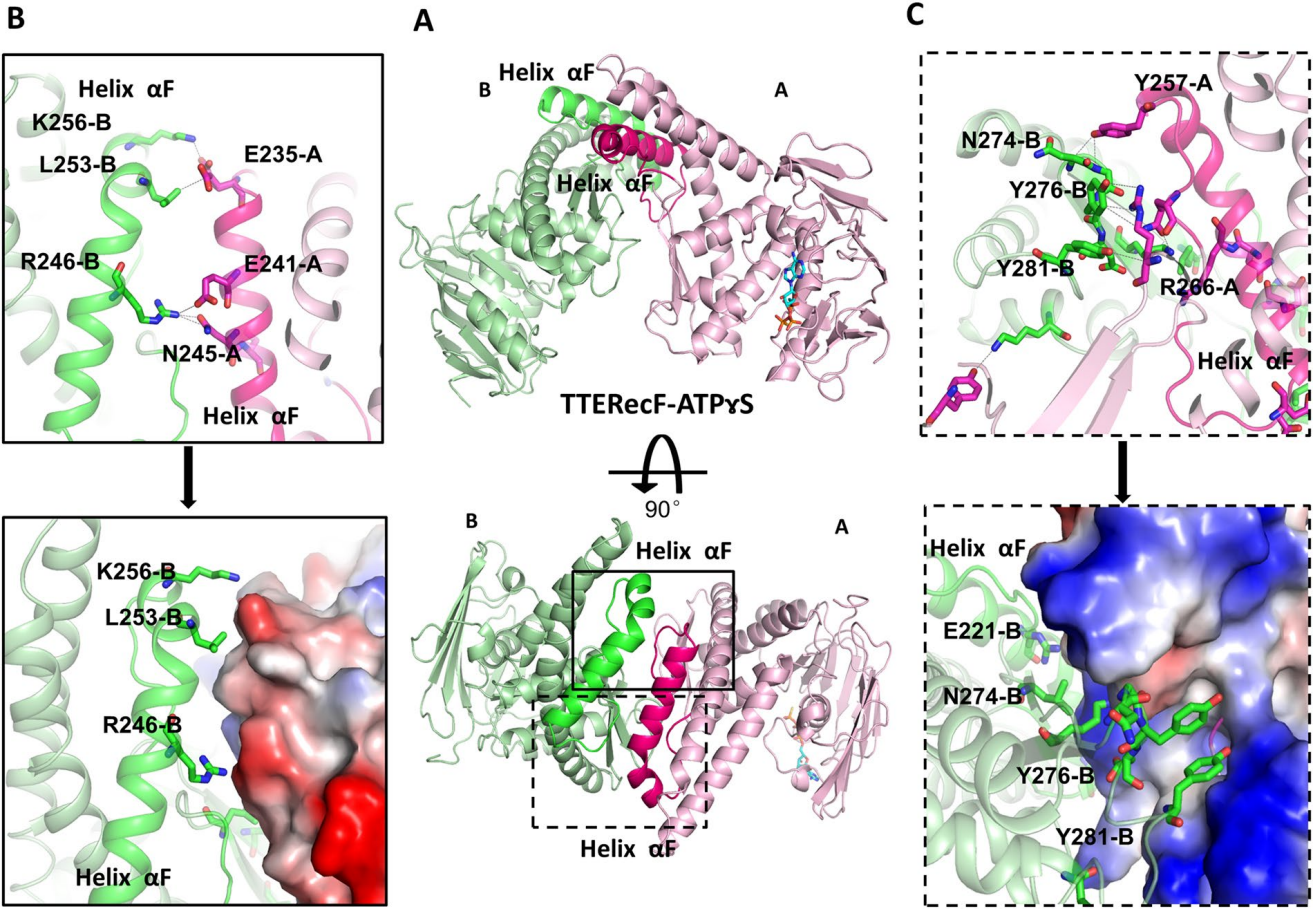
Interface of RecF-ATPrS dimer. **(A)** Cartoon representation of the RecF-ATPrS dimer (light pink and pale green), helix αF in monomer A is coloured hot pink, whereas helix αF in monomer B is light green. **(B,C)** Expanded view of the circle in Fig. 4A. Stick mode representation of the interacting amino acids in monomer B (light green) interact with amino acids (hot pink) in monomer A, or electrostatic properties of monomer A. Positive and negative potentials are shown in blue and red, respectively.

The structure of TTERecF-ATPɤS is also a dimer, that is completely different from the dimer of TTERecF-ATP and is thus another conformation of TTERecF (Fig. 5A). In the TTERecF-ATPɤS structure, domain I of the two monomers interacted, whereas their respective domain II structures were located far from each another (Supplementary Fig. 6 A). The dimer contact surface was 2613 Å^2^, which was 7.7% of the dimer total surface area (33774 Å^2^). The two monomers of TTERecF-ATPɤS were dissimilar; one monomer consisted of TTERecF-ATPɤS and the other consisted of TTERecF only. The Cα atoms of the two monomers was superimposed, with an r.m.s.d of 1.317 Å. One monomer structure (ATPɤS binding) was nearly similar to one monomer of TTERecF-ATP dimer, including the active site conformation of the Walker A motif, Walker B motif, and signature motif (Supplementary Fig. 6B). The Cα atoms of these two structures can be superimposed with an r.m.s.d of 0.807 Å. The other monomer structure (no ATPɤS binding) was similar to that of the free TTERecF (Supplementary Fig. 6C); the r.m.s.d of the superimposed Cα atoms was 1.121 Å.

According to the structure and interface of electrostatic potential, the interacting amino acids were mainly located in the RecF helix αF from the structure of TTERecF-ATPɤS (Figs. 5A–C). Then, we created a deletion mutant of RecF (229aa-262aa). This mutant appeared as s monomer in the solution. After incubation with 1 mM ATPɤS for 30 min, this deletion mutant remained as a monomer in the solution, as detected by size-exclusion chromatography (Supplementary Fig. 8 C). This result differed from the native RecF. Thus, we were sure that the RecF helix αF is the interface of the RecF-ATPrS dimer. Helix αF was conserved in the RecF protein (Supplementary Fig. 2), and it was missing in the Rad50 and SMC homologues. However, how the RecF-ATPɤS dimer was formed and how its conformation was altered to form ATP-mediated dimers upon DNA binding remained unknown. We speculated that the structure of the RecF-ATPrS dimer may be a physiological state or formed by crystal packing.

## Discussion

ITC assays indicated that the binding ability of TTERecF with ATPrS (*K*_D_ = 32 µM) was weaker than TTERecF with ATP (*K*_D_ = 1.3 µM) (Supplementary Fig. 8 A). In addition, the DNA binding affinities of the TTERecF-ATPɤS dimer for 21-mer ssDNA and dsDNA were 466 nM and 381 nM, which did not significantly differ from those of the native TTERecF (Supplementary Fig. 8B). The structural and biochemical data showed that the TTERecF-ATPɤS dimer cannot stably bind with DNA. The RecF ATP-driven dimerization induced the engagement of ABC domains and intersubunit rotations and presumably provided the major driving force for conformational switching. The TTERecF-ATP dimer structure displayed the closed reaction state of TTERecF for interacting with ATP and even for the complex interaction with DNA. Moreover, this reaction state is unique and stable.

RecF was a monomer in the solution; when the protein met ATP, the walker A motif of one RecF molecule served as a switch, and turned 180° to create a space for ATP binding. Subsequently, the signature motif of another TTERecF molecule interacted with ATP-Walker A motif to form a RecF-ATP dimer. ATP binding was less sensitive to the mutation of the signature motif; however, the signature motif was important for the dimerization and DNA-dependent ATP hydrolysis. DRRecF or ECRecF hydrolyzed negligible amounts of ATP^5,19^. We used an ATPase Assay Kit (Bioassay Systems) to check the ATPase activity of RecF; however, no ATPase activity was observed from TTERecF.

In the RecFOR pathway, the RecOR complex facilitated RecA filament formation on the SSB-coated ssDNA. The RecOR complex displayed a clear preference for ssDNA^16,17^. RecFOR, but not RecOR, was the most effective when RecF was bound near an ssDNA/ dsDNA junction^21^. Moreover, RecR interacted with RecF in the presence of ATP and DNA^18^. Therefore, the dimerization of RecF is important for DNA binding; ss/dsDNA junctional recognition; interactions with other protein partners, such as RecR, or a combination of these events.

## Materials and Methods

### Protein expression and purification

The*recF* (TTE0004; GenBank: AAM23321.1) was amplified from *T*. *tengcongensis* MB4 genomic DNA by PCR and individually cloned into the pETDuet plasmid (Novagen) for expression with an N-terminal hexahistidine tag. The proteins were overexpressed in *E*. *coli* BL21 (DE3). The cells were cultured in LB media containing 100 mg/l ampicillin at 37 °C for 8 h and induced with 0.4 mM isopropyl β-D-thiogalactoside (IPTG) for 10 h at 28 °C. The recombinant proteins were purified by sonication and two-step column chromatography using a Ni-affinity column and Superdex200 gel-filtration column (GE Healthcare). TTERecF was in a final buffer of the following composition: 20 mM Tris-Hcl, pH 7.0, 200 mM NaCl. TTERecF site-specific mutants were generated from the TTE-recF-pET Duet plasmid. All sequences were confirmed by sequencing. The mutants had the same purified method as the native TTERecF. All proteins were stored at −80 °C.

### Crystallization and data collection

The crystals of TTERecF and its complex with ATP or ATPɤS were obtained at 20 °C over a few days by the hanging drop vapor diffusion technique. TTERecF was crystallized in buffer containing 28% (w/v) PEG3350, 300 mM NaCl, 100 mM Tris-Hcl, pH 9.0. The crystals of TTERecF-ATP were obtained by 2 µL of TTERecF and 2 µL of reservoir solution (1.8 M ammonium sulfate, 100 mM Tris-Hcl, pH 8.5) with 3 mM ATP. The crystals of TTERecF- ATPɤS were obtained by 2 µL of TTERecF and 2 µL of reservoir solution (20% PEG3350, 300 mM NaCl, 100 mM Tris-Hcl, pH 7.0) with 3 mM ATPɤS. The crystals were flash-frozen by immersion in a reservoir of 15–25% glycerol followed by transferring to liquid nitrogen. The crystals were maintained at 100 K during X-ray diffraction data collection using the beamline BL17U and beamline BL19U (λ = 1.005 Å) at Shanghai Synchrotron Radiation Facility (SSRF; Shanghai, China). The diffraction images were indexed and integrated using HKL2000. The data collection statistics are presented in Table 1.

### Structure determination and refinement

The structure TTERecF was solved by the molecular replacement method using PHASER in the PHENIX suite^32^ with one monomer of DRRecF (PDB code: 2O5V) as the search model at 20-3 Å resolution. Iterative cycles of refinement and manual model building were carried out with PHENIX refinement programs and COOT, respectively, at 20-2.2 Å resolution. The structures of the TTERecF-ATP complex and TTERecF-ATPɤS were solved using the model of TTERecF, and refined using PHENIX refinement programs and COOT. All structural images were drawn using PyMOL (http://www.pymol.org/). Detailed crystallographic statistics are shown in Table 1.Coordinates have been deposited into PDB under the accession codes: 5Z67, 5Z68 and 5Z69.

### Size-exclusion chromatography

Size-exclusion chromatography was performed using a fast protein liquid chromatography system (GE Healthcare) on a Superdex-200 HR 10/300 column at a flow rate of 0.5 ml/min. The Native TTERecF (100 µM); TTERecF mutants G35A (10 µM), K36A (77 µM), S37A (35 µM), N38A (47 µM); TTERecF-ATP (ATP 1 mM) complex; TTERecF-ATPɤS (ATPɤS 2 mM) complex and TTERecF mutants-ATP (ATP 1 mM)complex were loaded onto the column equilibrated with 50 mMTris-HCl pH 7.0, 300 mM NaCl and eluted using the same buffer. Protein elution was monitored by measuring the absorbance at 280 nm and ATP elution was monitored by measuring the absorbance at 215 nm. Data analysis was conducted using UNICORN version 5.11 software program.

### Isothermal titration calorimetry (ITC)

ITC experiments were performed at a constant temperature of 25 °C using an ITC200 calorimeter (GE Life Science, MicroCal). Proteins and micromolecule were extensively dialysed against ITC buffer: 20 mM Tris-Hcl, pH 7.0, 200 mM NaCl. Protein concentrations were measured based on their respective ultraviolet absorption at 280 nm. The ITC experiments involved 20 injuections of 2 µL 1 mM micromolecule into 300 µL 70 µM protein. Reference measurements were carried out to compensate for the heat of dilution of the proteins. Experiments were repeated twice for each sample. The titration data were analyzed using the program Origin 7.0 and fitted by the one-site binding model.

### Biolayer interferometry (BLI) assays

The binding of ssDNA/dsDNA (5′-[Bio] ACCTTATGGAAAGCATCGTAG-3′) to TTERecF was measured by BLI using by Octet Red system (Ferbio). Streptavidin biosensors were hydrated in kinetics buffer (20 mM Tris-Hcl, pH 7.0, 200 mM NaCl, Tween 20 0.05%) at 25 °C for 10 min. After recording an initial baseline, the sensors were immersed in the solution of biotinylated DNA loading for 120 s. The Native TTERecF 100 µM and ATP 1 mM or ATPɤS 2 mM incubate for 30 min and then dilute to the specific concentration. Protein association (0.08 µM-5.12 µM TTERecF; 0.02 µM-0.64 µM TTERecF-ATP; 0.04 µM–2.56 µM TTERecF-ATPɤS;) for 120 s before sensors were washed and protein dissociation for 180 s. Subsequently, the biosensor was immersed in kinetics buffer to measure dissociation for 180 s. The *K*_D_ and *k*_d_ were calculated using the ForteBio Data Analysis 7.0 software. All images were drawn using Graph Pad Prism 5.

### Accession codes

Coordinates and structure factors have been deposited in the Protein Data Bank under accession codes 5Z67 for TTERecF, 5Z68 for TTERecF-ATP complex and 5Z69 for TTERecF-ATPɤS.

## Electronic supplementary material

Supplementary Dataset
