# Supplementary material for: ATP-dependent conformational change in ABC-ATPase RecF serves as a switch in DNA repair
Source: Sci Rep. 2018 Feb 1;8:2127. doi: 10.1038/s41598-018-20557-0 (PMC5794780; doi:10.1038/s41598-018-20557-0)
Supplement: Supplementary file 1 — Supplementary Dataset [file 41598_2018_20557_MOESM1_ESM.doc]

**ATP-dependent conformational change in ABC-ATPase RecF serves as switch in DNA repair**

Qun Tang, Yan-Ping Liu, Hai-Huan Shan, Li-FeiTian, Jie-Zhong Zhang & Xiao-Xue Yan

National Laboratory of Biomacromolecules, CAS Center for Excellence in Biomacromolecules, Institute of Biophysics, Chinese Academy of Sciences, Beijing 100101, China.

Correspondence should be addressed to Xiao-Xue Yan ( [snow@ibp.ac.cn](mailto:snow@ibp.ac.cn) )

**Supplemental Material**


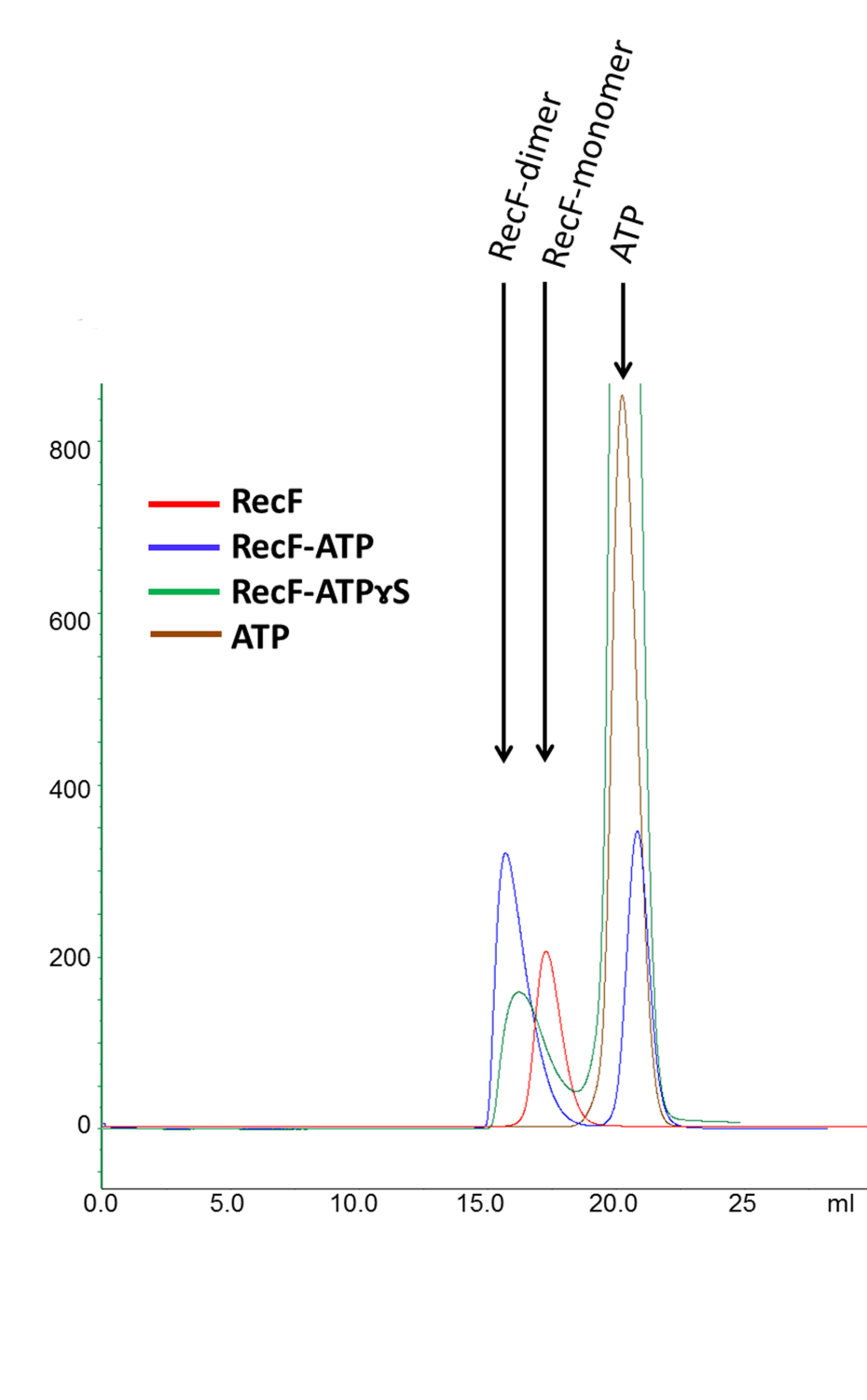


**Supplementary Figure 1.** Size-exclusion chromatography analysis of TTERecF, TTERecF-ATP and TTERecF- ATPɤS .


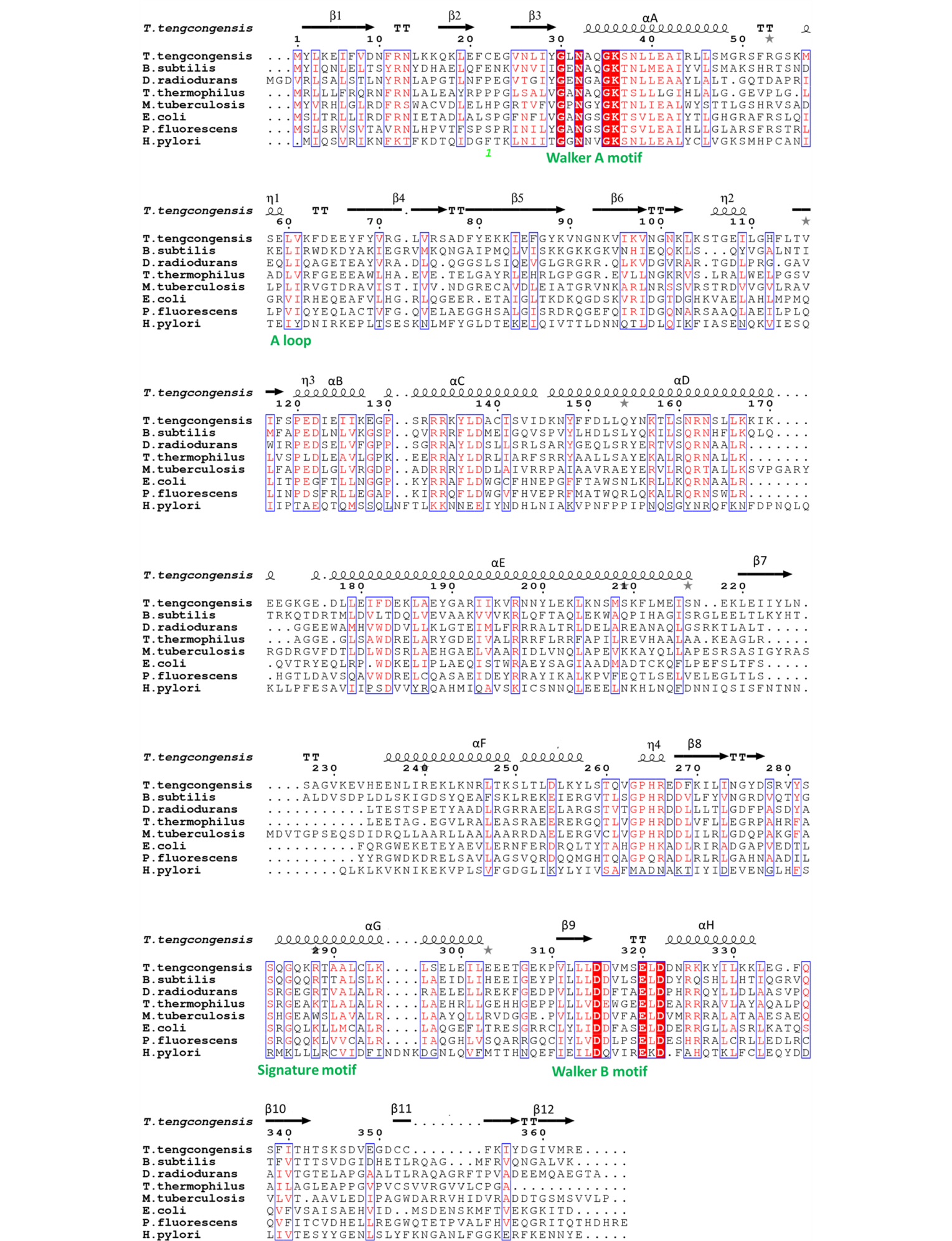


**Supplementary Figure 2. Sequence alignment of TTERecF and RecF from other species.** Sequence alignment was performed with ClustalX[^34^](#_ENREF_1). Strictly conserved residues are highlighted by red background and conservatively substituted residues are boxed. The figure was produced using ESPript[^35^](#_ENREF_2).


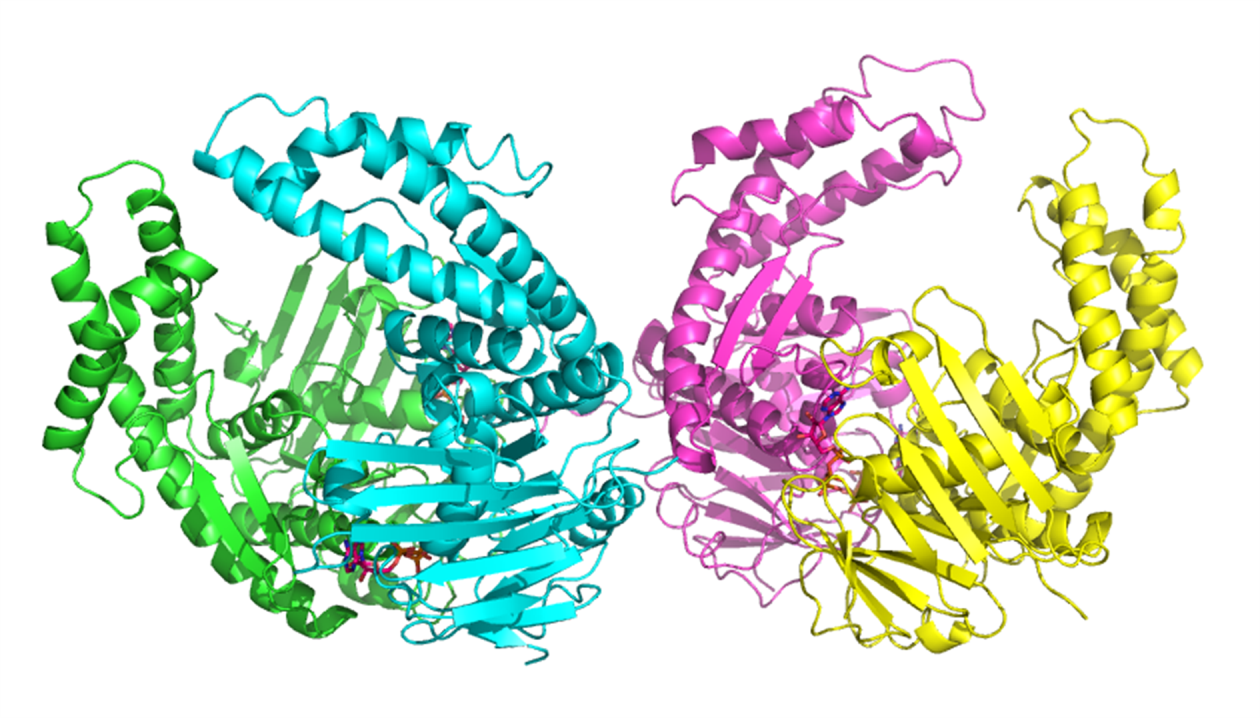


**Supplementary Figure 3. Crystal structure of RecF-ATP monomers in the asymmetric unit.** Four TTERecF-ATP complex monomers formed two dimers in the asymmetric unit. Each RecF-ATP shows in one color.


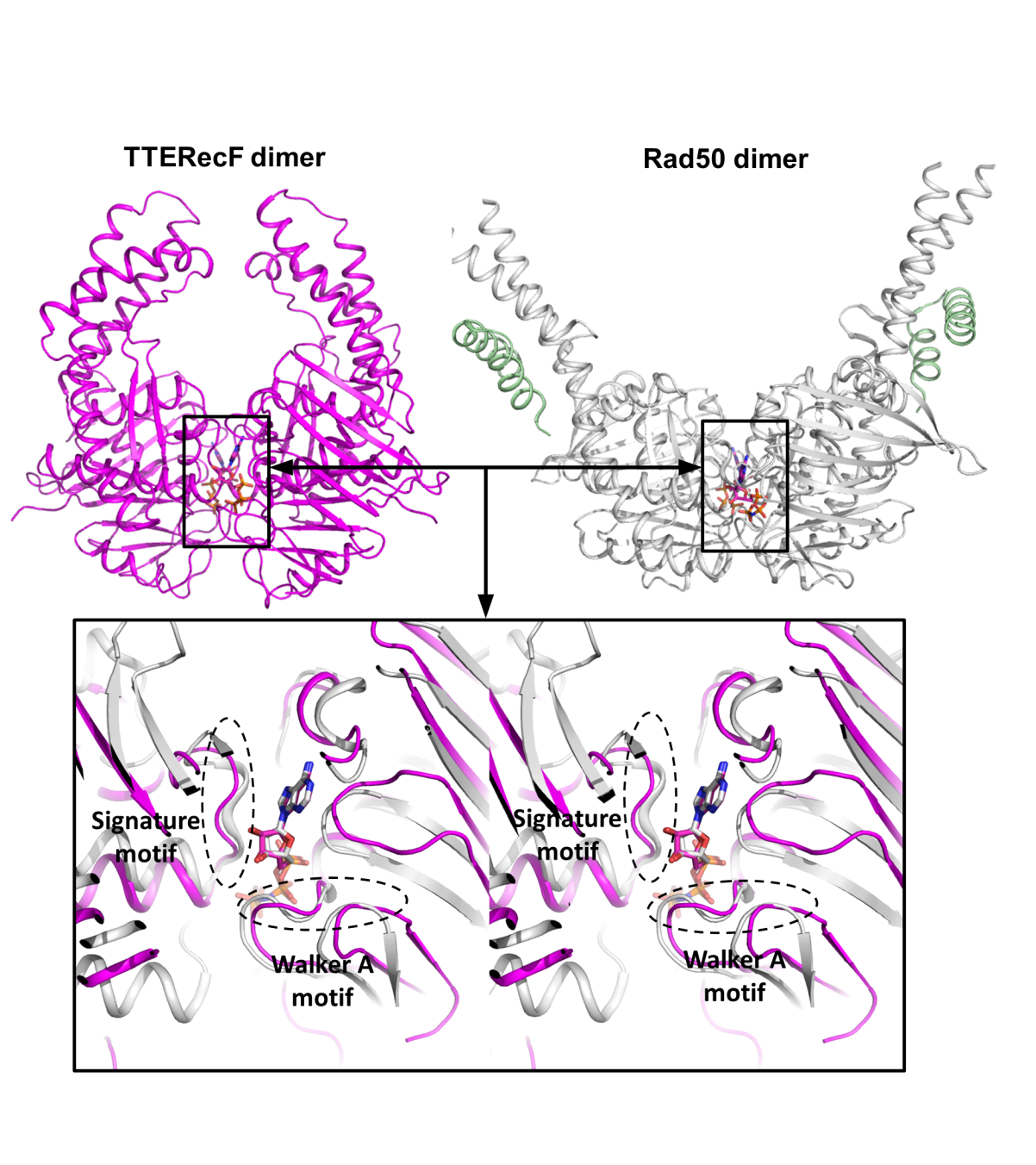


**Supplementary Figure 4. Comparison the structures of TTERecF-ATP dimer with Rad50 dimer.** Superimposed the ATP and the surrounding structures of RecF dimer and Rad50 dimerwall-eyed view showing the interactions between ATPand walker A motif and Walker B motif. ATP is shown as sticks.


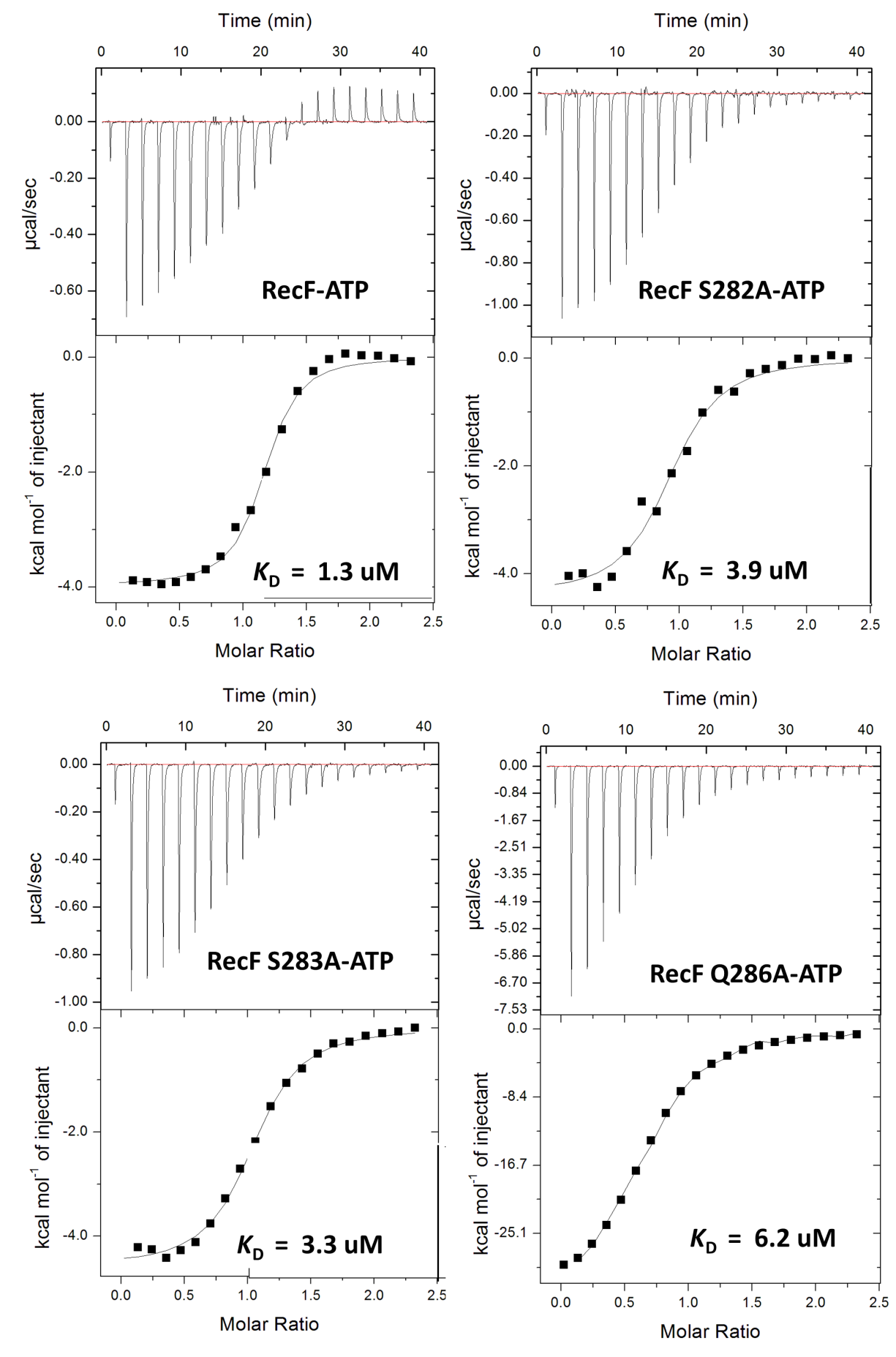


**Supplementary Figure 5.** **The ATP-binding ability of RecF and mutants**. ITC analysis of the dissociation constant (*K*_D_) of RecF and mutants interaction with ATP.


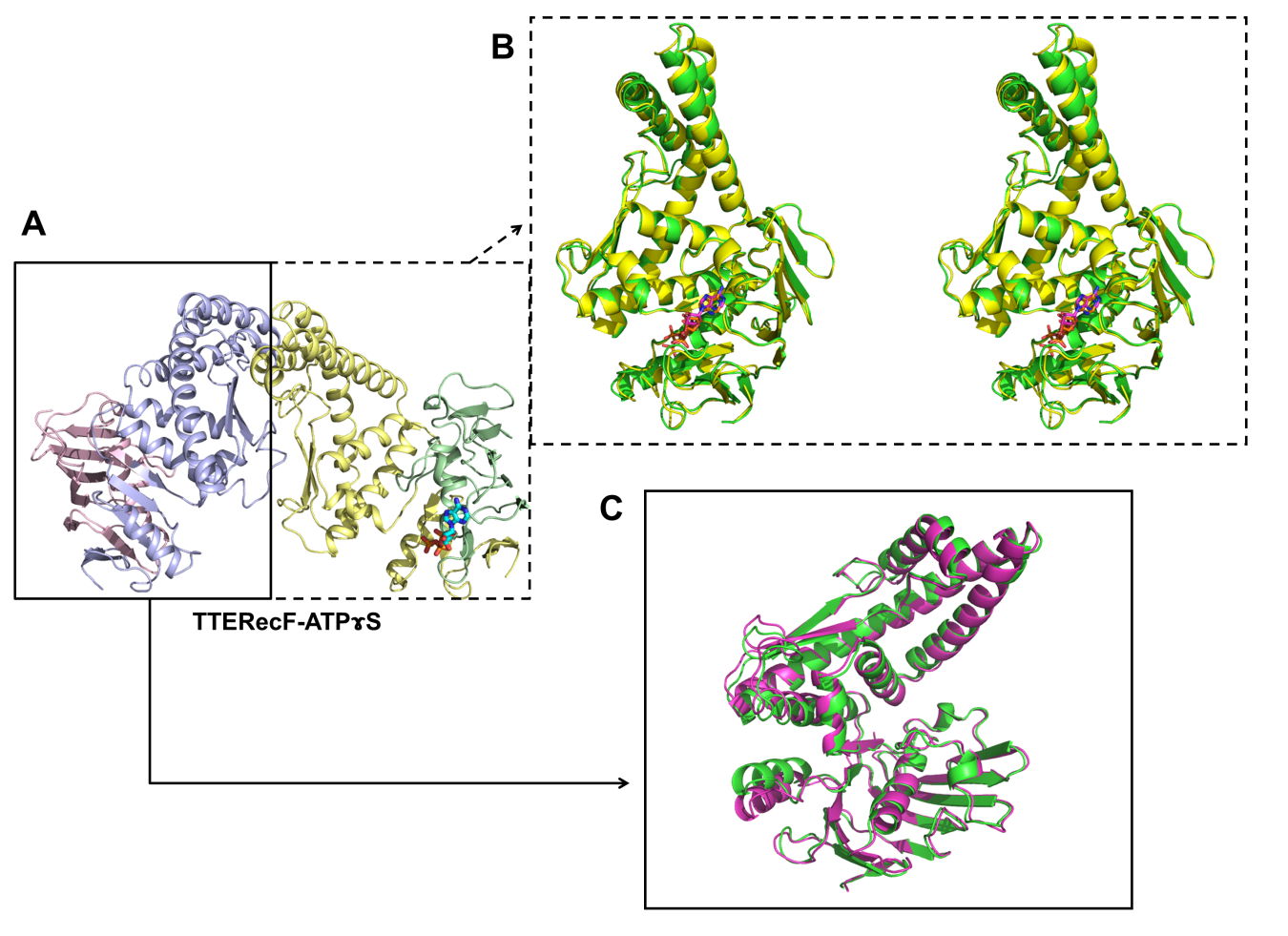


**Supplementary Figure 6. Crystal structure of TTERecF-ATPɤS.** (A) Crystal structure of TTERecF-ATPɤS.(B)Wall-eyed stereo view superimposed TTERecF-ATP (yellow) and TTERecF- ATPɤS (green) structures, TTERecF was shown as cartoon mood and ATP or ATPɤS were shown as sticks. (C) Superimposed TTERecF (purple) and TTERecF- ATPɤS (green) structures, TTERecF was shown as cartoon mood.


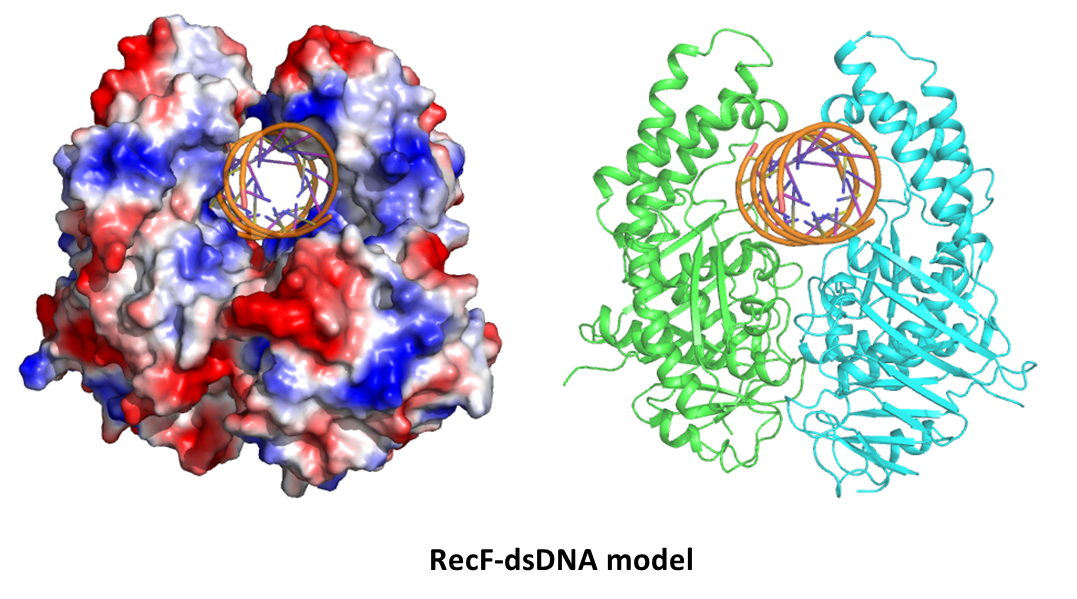


**Supplementary Figure 7. Structure model of RecF with dsDNA.** Electrostatic properties of TTERecF with dsDNA and cartoon mode of TTERecF with dsDNA.


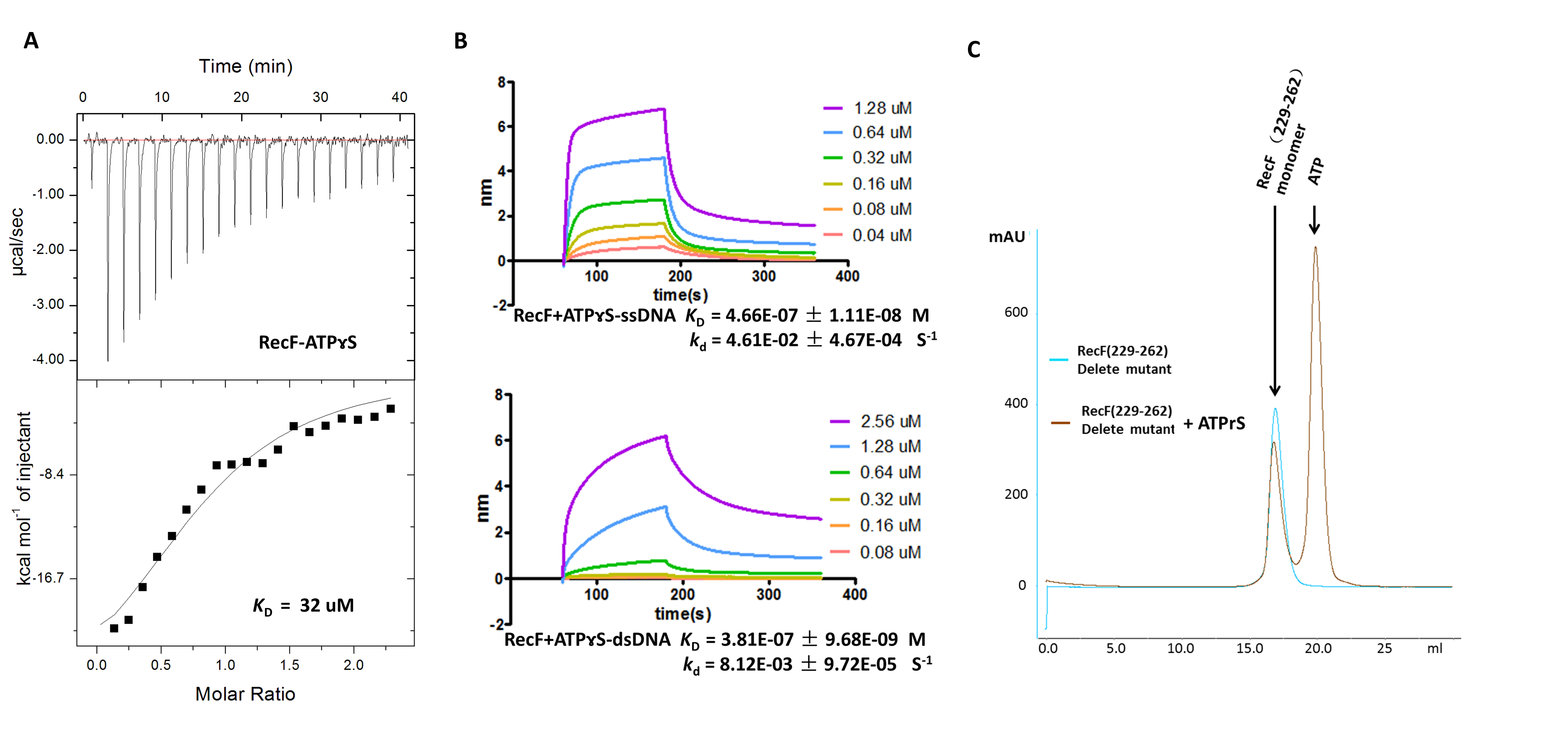


**Supplementary Figure 8. (A)** ITC analysis of the dissociation constant (*K*_D_) of RecF interaction with ATPrS

**(B)** BLI analysis of TTERecF-ATPɤS dimer interaction with ssDNA or dsDNA at 25 °C. **(C)** Size-exclusion chromatography analysis of TTERecF（229aa-262aa）deletion mutant with ATPrS.

34 Thompson, J. D., Gibson, T. J., Plewniak, F., Jeanmougin, F. & Higgins, D. G. The CLUSTAL_X windows interface: flexible strategies for multiple sequence alignment aided by quality analysis tools. *Nucleic Acids Res* **25**, 4876-4882 (1997).

35 Gouet, P., Courcelle, E., Stuart, D. I. & Metoz, F. ESPript: analysis of multiple sequence alignments in PostScript. *Bioinformatics* **15**, 305-308 (1999).
